# Effects of visual feedback balance training on muscle strength and balance ability in patients after anterior cruciate ligament reconstruction surgery

**DOI:** 10.1186/s12891-026-09823-9

**Published:** 2026-04-17

**Authors:** Xialin Ge, Mingxuan Gao, Longting Suo, Yiming Tao, Shuang Ren, Yingfang Ao

**Affiliations:** 1 Department of Sports Medicine, Peking University Third Hospital, Institute of Sports Medicine of Peking University, Beijing, 100191 China; 2 Beijing Key Laboratory of Research and Translation for Drugs and Medical Devices in Precision Diagnosis and Treatment of Sports Injuries, Beijing, 100191 China; 3 Engineering Research Center of Sports Trauma Treatment Technology and Devices, Ministry of Education, Beijing, 100191 China; 4 Tianjin Key Laboratory of Exercise Physiology and Sports Medicine, Institute of Sport, Exercise & Health, Tianjin University of Sport, Tianjin, 300381 China

**Keywords:** Anterior cruciate ligament reconstruction, Visual feedback, Balance training, Muscle strength, Rehabilitation

## Abstract

**Objective:**

To investigate the effects of visual feedback balance training on lower limb motor function in patients following anterior cruciate ligament reconstruction (ACLR), and to provide evidence for optimizing postoperative rehabilitation protocols.

**Design:**

Randomized controlled trial.

**Method:**

This study employed a prospective, single-blind, randomized controlled trial design. Initially, 42 patients were assigned (21 per group), with 33 ultimately completing the study (intervention group *n* = 17, control group *n* = 16). Both groups commenced a 10-week training program consisting of three sessions per week starting at week 5 postoperatively. Assessments were conducted preoperatively and postintervention using isokinetic muscle strength testing, dynamic balance testing, the Visual Analog Scale (VAS), the International Knee Disease Classification (IKDC) score, and the Lysholm score.

**Results:**

In terms of isokinetic strength of the thigh, the intervention group showed significant improvements in concentric quadriceps strength (Qc) and concentric hamstring strength (Hc) at 60°/s post-intervention (*P* < 0.05). In the control group, limb symmetry index (LSI) of Qc at 180°/s, as well as LSI of Hc at 60°/s and 180°/s, significantly decreased after the intervention (*P* < 0.05). Compared with the control group, the intervention group demonstrated significantly greater Qc and Hc at both 60°/s and 180°/s, 60°/s hamstring-to-quadriceps strength ratio (H/Q), and Qc and Hc LSI at both 60°/s and 180°/s (*P* < 0.05).Regarding dynamic balance, the intervention group showed significant improvements in green target performance, coordination, and stability (*P* < 0.05), significantly outperforming the control group across multiple metrics. The control group only showed improvement in coordination (*P* < 0.05). Regarding functional outcomes, the intervention group showed significant improvement in VAS and IKDC scores after the intervention (*P* < 0.05). However, improvement in VAS did not reach minimal clinically important difference (MCID) and was not clinically meaningful, whereas improvement in IKDC exceeded MCID. The intervention group also showed significantly greater improvements in VAS, IKDC, and Lysholm scores compared to the control group (*P* < 0.05).

**Conclusion:**

This preliminary study suggests that visual feedback balance training may improve lower limb muscle strength, dynamic balance, and functional outcomes in patients after ACLR, though these findings require confirmation in larger, long-term trials.

**Trial Registration:**

ClinicalTrials.gov (NCT07306221 registered on 20 November 2025 retrospectively registered).

## Introduction

Anterior cruciate ligament (ACL) injury is one of the most common severe knee injuries [1]. The ACL functions to restrict anterior tibial translation and maintain rotational stability of the knee joint [2]. Such injuries typically occur during high-impact activities involving sudden stops, changes of direction, or landing from jumps [3, 4] and are frequently seen in young athletes and physically active individuals [5]. Following injury, patients often experience knee instability and abnormal load distribution, accompanied by symptoms such as pain, swelling, and restricted motion. Secondary injuries to the meniscus and articular cartilage may also develop [6–8], accelerating joint degeneration and significantly impairing both athletic performance and quality of life [9, 10] .

ACLR is the preferred treatment for ACL injuries, effectively restoring the knee joint’s anatomical structure and stability [11]. However, surgery merely lays the foundation for functional recovery; postoperative rehabilitation training remains indispensable. Early intervention rehabilitation is particularly crucial for restoring lower limb muscle strength, balance, and motor function [12, 13]. Clinical practice indicates that even after conventional rehabilitation training, some patients persistently exhibit issues such as quadriceps-hamstring strength imbalance, dynamic instability of the lower limbs, and bilateral functional asymmetry [14–16]. These complications not only delay return to sports but may also increase the risk of re-injury and the incidence of secondary knee osteoarthritis [8]. Therefore, exploring more efficient postoperative rehabilitation protocols has become an urgent clinical priority. In recent years, the continuous advancement of biomechanics research has laid a solid theoretical foundation for optimizing rehabilitation strategies. Leveraging advanced technological methods, it has opened new avenues for enhancing athletic performance, reducing injury risks, and optimizing athlete rehabilitation [17].

Three-dimensional balance training utilizing visual feedback is a modern rehabilitation technique based on neuromuscular control theory. Through real-time visual feedback provided by a three-dimensional motion platform and diverse sensory stimuli in a dynamic spatial environment, it enables patients to intuitively perceive shifts in their body’s center of gravity and promptly adjust their posture. This approach effectively enhances balance, bilateral symmetry, and muscle strength [18–20]. Compared to traditional balance training, it offers advantages such as high specificity and immediate feedback. It has been preliminarily applied in rehabilitation for certain lower limb movement injuries and stroke [19, 21]. However, its application in early ACLR rehabilitation using a three-dimensional motion platform with real-time visual feedback has received limited investigation, particularly regarding its effects on lower limb isokinetic muscle strength, dynamic balance, and pain symptoms. Therefore, this study aims to investigate the effects of visual feedback balance training on lower limb function in patients following ACLR, with the goal of providing clinical evidence to optimize early postoperative rehabilitation protocols.

## Methods

### Participants

This study recruited patients undergoing unilateral anterior cruciate ligament reconstruction surgery at our hospital. Inclusion Criteria: (1) Patients aged 18 to 45 years with an anterior cruciate ligament rupture confirmed by MRI; (2) Patients who underwent arthroscopic ACL reconstruction using a unilateral autologous hamstring tendon graft; (3) Patients willing to adhere to the rehabilitation protocol and complete the training program; (4) Absence of injury to the posterior cruciate ligament (PCL) or lateral collateral ligament (LCL). Injury to the medial collateral ligament (MCL), if present, was limited to grade I (mild sprain/elongation with normal MRI signal).Exclusion Criteria: (1) Body mass index (BMI) < 18.5 or > 35 kg/m²; (2) Age < 18 or > 45 years; (3)Concurrent knee pathologies, such as osteoarthritis, tumor, inflammatory arthritis (e.g., rheumatoid arthritis), or tuberculosis; (4) Cognitive impairment; (5) Inability to complete the rehabilitation training or outcome assessments.

Participants signed informed consent forms after understanding the detailed experimental procedures. The use of human subjects in this study was approved by the institutional review board and registered at the US Clinical Trials Registry (NCT07306221). Each participant signed a written consent form before any data were collected. No significant differences existed between the two groups in baseline demographic characteristics (*P* > 0.05); thus, they were comparable (Table 1). The study adhered to the ethical principles outlined in the 1964 Helsinki Declaration and its subsequent amendments. This study followed the CONSORT guidelines for reporting randomized controlled trials. No adverse events occurred in either group during the training and evaluation process.

**Table 1:** Characteristics of participants (*M* ± *SD*)

Characteristic	Intervention group (*n* = 17)	Control group (*n* = 16)	*P* value
Age (years)	28.35 ± 6.13	30.00 ± 7.87	0.506
Height (m)	1.76 ± 0.07	1.73 ± 0.08	0.435
Body mass (kg)	77.18 ± 9.10	75.80 ± 10.61	0.690
Body mass index (kg/m^2^)	24.93 ± 2.47	25.02 ± 2.38	0.915
Affected side (left/right)	10/7	11/5	

### Sample size

The International Knee Documentation Committee (IKDC) score was used as the observation index. Based on the pre-experiment results, according to the calculation formula [22]:$\:{n}_{2}=\frac{({z}_{1-\alpha\:/2}+{z}_{1-\beta\:}{)}^{2}(s{d}_{1}^{2}+s{d}_{2}^{2}\left)\right(1+1/k)}{2(mea{n}_{1}-mea{n}_{2}{)}^{2}}$, $\:{n}_{1}=k\times\:{n}_{2}$, $\:\mathrm{w}\mathrm{h}\mathrm{e}\mathrm{r}\mathrm{e}{\:\mathrm{n}}_{1}$ and $\:{\mathrm{n}}_{2\:}$ represent the required sample size for each group, K represents the 1:1 allocation ratio ; $\:{\mathrm{z}}_{1-{\upalpha\:}/2}$=1.96 is the critical value for a two-sided Type I error ($\:{\upalpha\:}=0.05$), and $\:{\mathrm{z}}_{1-{\upbeta\:}}$=0.84 is the critical value for a Type II error ($\:{\upbeta\:}=0.20$, corresponding to 80% statistical power). The standard deviations for the intervention and control groups were $\:\mathrm{s}{\mathrm{d}}_{1}=2.89$ and $\:\mathrm{s}{\mathrm{d}}_{2}=14.78\:$, respectively, and the group means were $\:{\mathrm{m}\mathrm{e}\mathrm{a}\mathrm{n}}_{1}=70.50$ and$\:{\:\mathrm{m}\mathrm{e}\mathrm{a}\mathrm{n}}_{2}=57.90$. Based on these values, we calculated that a sample size of 10 participants per group would achieve 80% statistical power at a two-sided $\:{\upalpha\:}=0.05\:$to detect a mean difference of 12.60 points (70.50–57.90) between the two groups. Considering an anticipated 20% dropout rate, the final adjusted sample size was 13 participants per group.

### Grouping and blinding design

A single-blind randomized controlled trial design was adopted in this study. A rehabilitation therapist who was not involved in participant recruitment, baseline assessment or outcome evaluation generated a random sequence using a computerized random number table to ensure the unbiased allocation of participants to each intervention group. Group assignment information was placed in sequentially numbered, sealed and opaque envelopes. Participants were unaware of their group assignments after completing the baseline assessment and before the envelopes were opened by the intervention implementers, thus ensuring the implementation of allocation concealment. Due to the inherent nature of the exercise intervention, blinding of participants was not feasible. However, outcome assessors and statistical analysts were blinded in this study, meaning that the above personnel remained unaware of the participants’ group assignments throughout the study, so as to minimize the risks of measurement bias and outcome interpretation bias. To ensure sufficient test efficiency even if the dropout rate was higher than expected, we initially recruited 21 participants per group (a total of 42 participants). During the study, there were 9 dropouts (4 in the intervention group and 5 in the control group), with a final total of 33 participants (17 in the intervention group and 16 in the control group) included in the final analysis.

### Intervention methods

After enrollment, participants in both the intervention and control groups completed routine rehabilitation therapy during the first 4 weeks after surgery. From postoperative weeks 5 to 14, all participants followed a 10-week supervised exercise program consisting of three sessions per week, each lasting approximately 70–80 min. Each session began with 3–5 min of warm up and ended with 3–5 min of stretching and relaxation. Ice was applied as needed after training. In addition to the conventional rehabilitation exercises, the control group performed standard balance training, whereas the intervention group performed visual feedback balance training. During supervised training, researchers assisted participants in refining their movement techniques through verbal guidance and manual correction.

**Fig. 1:**
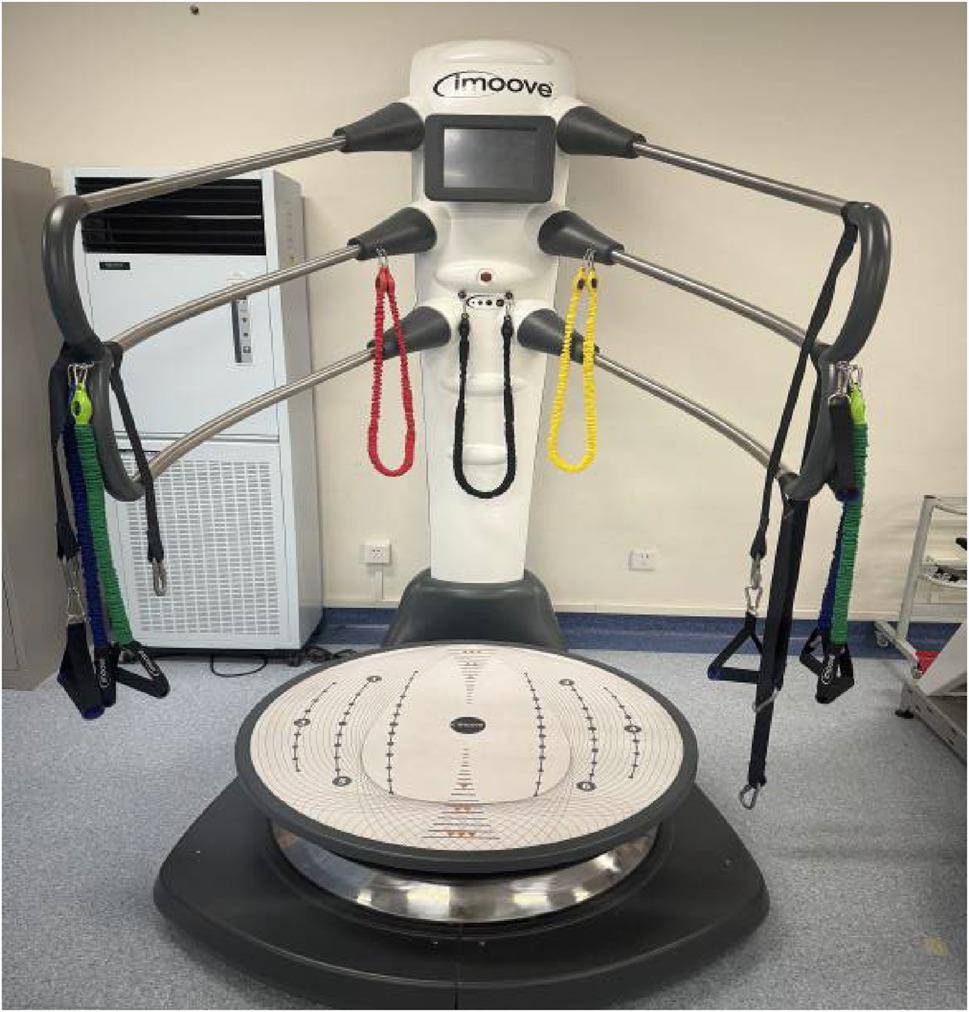
Imoove 500 Balance System

- (1) Conventional Rehabilitation Training
  - During weeks 0–4 after surgery, exercises included ankle pumps, quadriceps setting, hamstring setting, straight leg raises, passive knee flexion, and passive knee extension.
  - During weeks 5–14, exercises comprised passive knee flexion/extension, straight leg raises, multidirectional leg lifts (posterior, lateral, medial), double and single leg bridging on a mat, and limb strengthening exercises.Training volume was 1 h per day, 3 days per week, for 10 weeks.
- (2) Standard Balance Training
  - Balance exercises included forward lunges, backward lunges, double leg squats, single leg stance, and single leg stepping forward/backward. Exercise difficulty was progressed based on the patient’s recovery. Training was performed for 20 min per day, 3 days per week, over 10 weeks.
- (3) Visual Feedback Balance Training 
  - Visual feedback balance training was conducted using the Imoove 500 Balance System (Version 2.1, Allcare Innovation, Lourdes, France) with the knee training module（Figure 1). Training amplitude and speed were adjusted every 2 weeks. From weeks 5 to 10 after surgery, the training focused on five main movements (e.g., squat to stand, weight shifting, and lunge exercises). From weeks 11 to 14, the protocol included five movements such as squat to stand, single leg stance, and lunge exercises. Each session lasted 20 min, 3 days per week, for a total of 10 weeks.

### Evaluation indicators

#### Isokinetic strength

The isokinetic force test was performed using an isokinetic dynamometer (CON-TREX MJ, version MK2m, Physiomed Elektromedizin AG, Schnaittach, Germany) at a sampling frequency of 256 Hz. Participants were seated on the device with straps stabilizing the trunk and the tested knee. The range of motion was set from 20° to 90° of knee flexion. The strength was evaluated at angular velocities of 60°/s and 180°/s in concentric mode. After a gravity-correction procedure and 1–2 practice trials, participants performed 5 maximal repetitions of knee flexion and extension. The unaffected limb was tested first, followed by the affected limb. Isokinetic strength testing has been demonstrated to be a reliable predictor of functional performance and athletic capacity in various populations [23]. The following parameters were recorded: relative peak torque of the knee extensors and flexors, the hamstring-to-quadriceps peak torque ratio (H/Q), and the Limb Symmetry Index (LSI).

$$\:\mathrm{L}\mathrm{S}\mathrm{I}=\mathrm{i}\mathrm{n}\mathrm{j}\mathrm{u}\mathrm{r}\mathrm{e}\mathrm{d}\:\mathrm{l}\mathrm{e}\mathrm{g}/\mathrm{u}\mathrm{n}\mathrm{i}\mathrm{n}\mathrm{j}\mathrm{u}\mathrm{r}\mathrm{e}\mathrm{d}\:\mathrm{l}\mathrm{e}\mathrm{g}\times\:100\mathrm{\%}$$

#### Dynamic balance ability

Dynamic balance was assessed using the Imoove 500 balance system (Version 2.1, Allcare Innovation, Lourdes, France). The system offers three difficulty levels (low, medium, extreme), each with three sensitivity settings. Based on pilot results and expert consensus, the low-difficulty and low-sensitivity setting was used for formal evaluation. Participants stood on the three-dimensional motion platform in a slightly squatted position with the core engaged to maintain stability. During the test, they focused on the screen while holding hand controllers, attempting to keep an on-screen cursor within a target area for 1 min. Participants completed a full warm-up and familiarization trial before formal testing. The assessed metrics included: Green Target Score, Coordination Test, Imbalance Percentage, Stability Test, Balance Distribution, and Overall Score (a composite of the first five items). Higher scores represent better performance.

#### Functional outcomes

Functional outcomes were assessed using the following instruments: Visual Analogue Scale (VAS) [24]: Pain intensity was rated on a 10-cm horizontal line, where “0” represents “no pain” and “10” represents “the worst pain imaginable.” Patients marked the point corresponding to their current level of pain. The IKDC [25] Subjective Knee Form includes items on symptoms, function, and sports activities. Scores range from 0 to 100, with higher scores indicating better knee function. Lysholm Knee Score [26]: This scale assesses eight domains: limping, use of support, locking, instability, pain, swelling, stair climbing, and squatting. The total score ranges from 0 to 100, with higher scores reflecting better functional status. It is commonly used to monitor recovery after knee injury or surgery. The minimum clinically significant difference (MCID) of VAS was set to 1.5 [27], IKDC to 9 and Lysholm to 10 [28], which were used to evaluate the clinical significance of improvement.

### Statistical analysis

Statistical analyses were performed using SPSS version 27.0 (IBM Corp, Armonk, NY, USA). The normality of continuous variables was assessed using the Shapiro-Wilk test. For intra-group comparisons, normally distributed continuous data are expressed as mean ± standard deviation (SD) and analyzed using paired-sample t-tests, with effect sizes (ES) calculated and interpreted as small (≥ 0.2 and < 0.5), moderate (≥ 0.5 and < 0.8), and large (≥ 0.8) effects [29]. For continuous variables not normally distributed, data are presented as median (interquartile range) and analyzed using the Wilcoxon signed-rank test; effect sizes were calculated as r values in post hoc analyses, with r values of 0.10–0.29, 0.30–0.49, and ≥ 0.50 corresponding to small, medium, and large effects, respectively [30]. For comparisons of baseline values between groups, an independent samples t-test was used for normally distributed variables, and a Mann-Whitney U test was used for non-normally distributed variables. Between-group differences were assessed using analysis of covariance (ANCOVA), with post-intervention values as the dependent variable, group as a fixed factor, and baseline values as covariates to adjust for baseline differences. Interactions between group and covariates were examined to assess the homogeneity of regression slopes. When assumptions were satisfied, Bonferroni correction was applied for multiple comparisons. Effect sizes were reported as partial eta squared (η²), with values of 0.01, 0.06, and 0.14 representing small, medium, and large effects, respectively [31]. A two-tailed *P* value of less than 0.05 was considered statistically significant.

## Results

### Isokinetic muscle strength test

Statistical results show that, in within-group comparisons, the intervention group demonstrated significant improvements in 60 °/s Qc and 60 °/s Hc compared to baseline (Cohen’s d = 0.897, *P* = 0.002; Cohen’s d = 0.939, *P* = 0.001); in the control group, the 60 °/s Hc LSI was significantly lower after the intervention than at baseline (Cohen’s d = 0.617, *P* = 0.026). Results from the between-group comparison showed no significant differences in baseline values for any of the indicators between the two groups (*P* > 0.05), indicating that the two groups were comparable. Analysis of covariance (ANCOVA) revealed that, after adjusting for baseline, the intervention group’s values for 60 °/s Qc, 60 °/s Hc, 60 °/s H/Q, 60 °/s Qc LSI, and 60 °/s Hc LSI were significantly superior to those of the control group (*F* = 10.758, *P* = 0.003, η_p_^2^ = 0.271; *F* = 18.849, *P* < 0.001, η_p_^2^ = 0.394; *F* = 5.232, *P* = 0.030, η_p_^2^ = 0.153; *F* = 10.379, *P* = 0.003, η_p_^2^ = 0.263; *F* = 11.758, *P* = 0.002, η_p_^2^ = 0.288). (Table 2)

**Table 2:** Changes in isokinetic concentric 60°/s parameters of the knee joint

Items	Group	Baseline	Post-intervention	ANCOVA F	*P* value	Partial η²
Qc, Nm/kg	Intervention Group	1.37 ± 0.54	1.68 ± 0.51^a^	10.758	0.003^b^	0.271
	Control Group	1.05 ± 0.51	0.97 ± 0.56			
Hc, Nm/kg	Intervention Group	0.85 ± 0.31	1.07 ± 0.25^a^	18.849	< 0.001^b^	0.394
	Control Group	0.75 ± 0.35	0.69 ± 0.25			
H/Q	Intervention Group	0.64 ± 0.11	0.65 ± 0.13	5.232	0.030^b^	0.153
	Control Group	0.72 ± 0.13	0.84 ± 0.36			
Qc LSI, %	Intervention Group	79.65 ± 25.78	83.76 ± 17.42	10.370	0.003^b^	0.263
	Control Group	76.38 ± 22.62	61.86 ± 27.64			
Hc LSI, %	Intervention Group	86.46 ± 19.85	89.00 ± 10.32	11.758	0.002^b^	0.288
	Control Group	82.94 ± 22.59	68.40 ± 19.11^a^			

*Qc* concentric quadriceps strength, *Hc* concentric hamstring strength, *H/Q* Hamstring to Quadriceps Strength Ratio, *LSI* Limb Symmetry Index

^a^ Within-group comparison, *P* < 0.05; ^b^ Between-group comparison, *P* < 0.05

Statistical results show that, in within-group comparisons, the 180°/s Qc LSI and 180°/s Hc LSI in the control group decreased significantly from baseline levels following the intervention (Cohen’s d = 0.667, *P* = 0.016; Cohen’s d = 0.878, *P* = 0.003). In the between-group comparison, there were no significant differences in baseline values for the 180°/s isokinetic concentric exercise metrics between the two groups (*P* > 0.05). Analysis of covariance (ANCOVA) revealed that, after adjusting for baseline, the intervention group showed significantly greater improvements in 180°/s Qc, 180°/s Hc, 180°/s Qc LSI, and 180°/s Hc LSI compared to the control group (*F* = 12.838, *P* = 0.001, η_p_^2^ = 0.307; *F* = 11.304, *P* = 0.002, η_p_^2^ = 0.280; *F* = 8.059, *P* = 0.008, η_p_^2^ = 0.217; *F* = 9.432, *P* = 0.005, ηp^2^ = 0.245). (Table 3)

**Table 3:** Changes in isokinetic concentric 180°/s parameters of the knee joint

Items	Group	Baseline	Post-intervention	ANCOVA F	*P* value	Partial η²
Qc, Nm/kg	Intervention Group	1.14 ± 0.48	1.19 ± 0.39	12.838	0.001^b^	0.307
	Control Group	1.03 ± 0.47	0.75 ± 0.37			
Hc, Nm/kg	Intervention Group	0.77 ± 0.27	0.82 ± 0.23	11.304	0.002^b^	0.280
	Control Group	0.74 ± 0.29	0.57 ± 0.20			
H/Q	Intervention Group	0.70 ± 0.14	0.73 ± 0.24	2.139	0.154	0.069
	Control Group	0.74 ± 0.15	0.87 ± 0.40			
Qc LSI, %	Intervention Group	83.46 ± 19.86	77.81 ± 24.83	8.059	0.008^b^	0.217
	Control Group	84.82 ± 26.38	58.63 ± 23.65^a^			
Hc LSI, %	Intervention Group	90.98 ± 0.21	86.57 ± 11.91	9.432	0.005^b^	0.245
	Control Group	90.82 ± 24.01	68.52 ± 21.59^a^			

*Qc* concentric quadriceps strength, *Qc* concentric quadriceps strength, *Hc* concentric hamstring strength, *H/Q* Hamstring to Quadriceps Strength Ratio, *LSI* Limb Symmetry Index

^a^ Within-group comparison, *P* < 0.05; ^b^ Between-group comparison, *P* < 0.05

### Dynamic balance ability test

The intervention group showed significant improvements in green target scores, coordination, imbalance percentage, and total scores post-intervention (Cohen’s d = 0.716, *P* = 0.003; Cohen’s d = 0.731, *P* = 0.003; Cohen’s d = 0.747, *P* = 0.002; Cohen’s d = 0.861, *P* < 0.001). The control group showed significant improvement in coordination after the intervention (Cohen’s d = 0.544, *P* = 0.030). The intervention group demonstrated significantly superior post-intervention scores in green target performance, imbalance percentage, and total score compared to the control group (Cohen’s d = 0.440, *P* = 0.011; Cohen’s d = 0.361, *P* = 0.038; Cohen’s d = 0.562, *P* = 0.001). (Table 4)

**Table 4:** Comparison of dynamic balance capabilities

Items	Group	Baseline	Post-intervention	Effect Size (Cohen’s d)	*P* value
Green Target Performance	Intervention Group	86.0(18.0)	97.0(4.5)	0.716	0.003^a^
	Control Group	76.0(35.5)	86.5(12.8)^b^	0.414	0.098
Coordination	Intervention Group	87.0(9.5)	90.0(3.0)	0.731	0.003^a^
	Control Group	84.0(34.0)	86.0(14.0)	0.544	0.030^a^
Imbalance percentage	Intervention Group	98.0(3.0)	100.0(2.0)	0.747	0.002^a^
	Control Group	98.0(6.0)	98.0(7.5)^b^	0.105	0.675
Stability	Intervention Group	53.0(7.9)	57.0(12.0)	0.389	0.109
	Control Group	57.5(6.0)	56.0(13.8)	0.052	0.836
Balanced Distribution	Intervention Group	89.0(3.0)	93.0(6.0)	0.364	0.133
	Control Group	87.5(7.5)	91.0(5.8)	0.448	0.073
Total Score	Intervention Group	83.2(6.3)	86.8(2.5)	0.861	< 0.001^a^
	Control Group	80.0(13.7)	83.4(3.9)^b^	0.469	0.061

^a^ Within-group comparison, *P* < 0.05; ^b^ Between-group comparison, *P* < 0.05;

### Functional outcome assessment

**Fig. 2:**
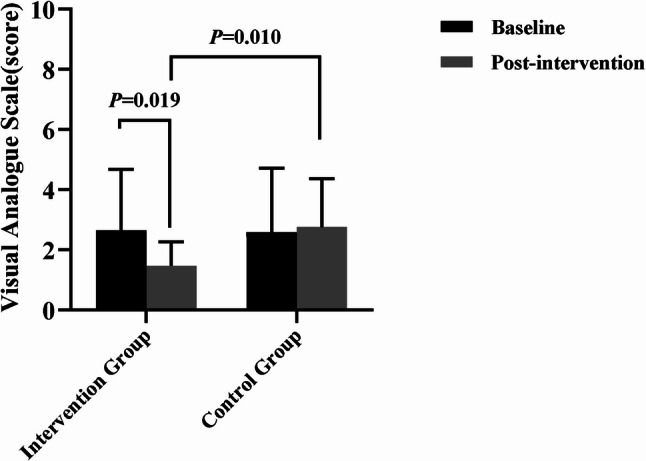
Comparison of VAS score results

**Fig. 3:**
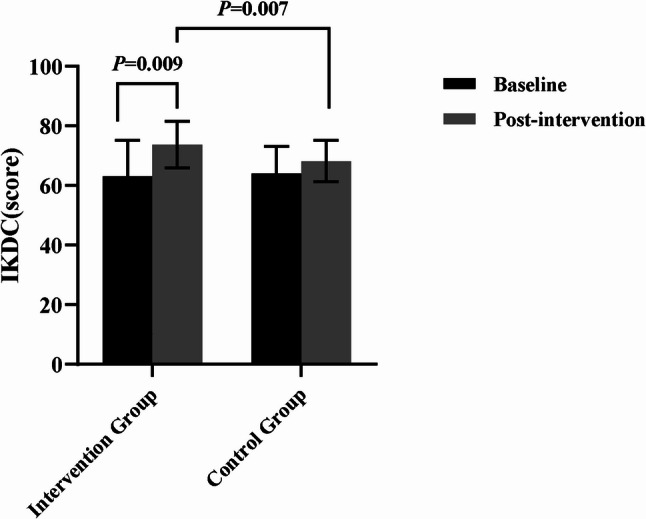
Comparison of IKDC scoring results

**Fig. 4:**
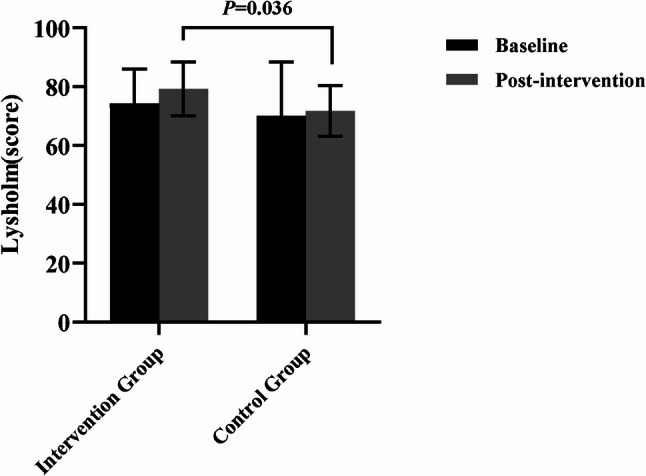
Comparison of lysholm scoring results

The statistical results showed that after the intervention, the VAS score improved by an average of 1.18 points (Cohen’s d *=* 0.570, *P* = 0.019) in the intervention group, which did not reach the MCID, and the IKDC score improved by an average of 10.53 points (Cohen’s d = 0.726, *P* = 0.009), which exceeded the MCID. There was no significant difference in baseline comparison between the two groups (*P* > 0.05). The results of covariance analysis showed that the improvement of VAS, IKDC and Lysholm scores in the intervention group was significantly better than that in the control group (*F* = 7.588, *P* = 0.010, η_p_^2^ = 0.207; *F* = 0.526,*P* = 0.007,η_p_^2^ = 0.227; *F* = 4.814, *P* = 0.036, η_p_^2^ = 0.142), and the differences were statistically significant.(Figures 2 and 3, and 4).

IKDC, International Knee Documentation Committee Subjective Knee Form.

## Discussion

This randomized controlled trial systematically evaluated the effects of visual-feedback balance training on lower limb motor function in patients after ACLR. The results indicate that, compared with traditional balance training, visual-feedback balance training led to significantly greater improvements in muscle strength, dynamic balance, pain reduction, and patient-reported functional outcomes. These findings support the integration of visual-feedback training into early postoperative rehabilitation protocols following ACLR.

After ACLR, persistent muscle weakness and bilateral lower limb asymmetry remain major barriers to functional recovery [32–35]. The results of this study indicate that visual feedback balance training can significantly improve lower limb muscle strength and symmetry in the intervention group, whereas conventional rehabilitation may have certain limitations in maintaining or improving muscle symmetry. Further between-group comparisons suggest that this training approach may promote lower limb muscle strength recovery and optimize limb symmetry across different angular velocities. From a functional perspective, low angular velocity (60°/s) primarily reflects maximal muscle strength, whereas high angular velocity (180°/s) better represents rapid force production and dynamic performance. The improvements observed in the intervention group at high angular velocity suggest enhanced neuromuscular responsiveness and regulation during dynamic tasks, which are critical for functional activities such as running and cutting movements, and may also reflect improvements in dynamic balance. From a potential mechanistic perspective, patients with ACL injury or following ACLR often rely more heavily on visual information to compensate for impaired balance control [36]. Visual feedback balance training provides real-time postural information, which may facilitate the integration of vestibular, visual, and proprioceptive inputs within the central nervous system, thereby enhancing lower limb motor control and dynamic trunk stability [37, 38]. Moreover, this training, conducted on an elliptical three-dimensional motion platform, requires participants to continuously adjust their center of mass and posture during movement [39], thereby activating periarticular proprioceptors around the knee and optimizing neuromuscular control processes [40, 41], consistent with the findings of Fitzgerald et al. [42]. Furthermore, visual feedback, as an exogenous information input, may enhance the coupling between sensory input and motor output, promote adaptive remodeling of the central nervous system, improve information processing efficiency, and refine sensorimotor integration [43, 44].With continued training, the transmission efficiency of visual input and proprioceptive signals gradually increases, and the ability of the cerebral cortex to integrate environmental, sensory, and motor information may be enhanced. In this process, the gradual increase in task difficulty further facilitates the recruitment of periarticular muscles, postural control, and dynamic stability [36, 45]. Notably, this study employed phased adjustments of training parameters to achieve individualized progressive load control, which helps prevent suboptimal rehabilitation outcomes caused by inadequate loading while also reducing the risk of overtraining. In contrast, conventional balance training lacks objective feedback and appropriate load modulation, with patients primarily relying on self-regulation to maintain stability [46], which may lead to inefficient muscle activation and affect the recovery of lower limb muscle strength due to compensatory activation patterns. Previous meta-analytic evidence indicates that task-specific, feedback-driven training programs confer greater advantages over traditional resistance training in improving neuromuscular control and strength adaptations [47]. Therefore, visual feedback balance training not only facilitates lower limb strength recovery and symmetry improvement but may also enhance sensorimotor integration and dynamic stability, thereby potentially reducing the long-term risk of reinjury. However, these mechanisms remain to be fully validated, particularly through multimodal evaluations combining neuroimaging and kinematic analyses to further elucidate the underlying neurophysiological mechanisms.

Dynamic balance ability is essential for maintaining knee stability during movement, and its insufficient recovery remains a significant contributor to functional limitations after ACLR. Evidence suggests that enhancements in balance are closely associated with improved periarticular muscle function and limb control [48]. In the present study, the intervention group demonstrated significant post-intervention improvements in green target performance, coordination, imbalance percentage, and total score, gains that were substantially greater than those observed in the control group, which improved only in coordination. This result fully confirms that visual feedback balance training is more effective than traditional training methods in enhancing dynamic balance ability and postural control in patients after ACLR surgery. However, it should be objectively noted that the intervention group underwent 10 weeks of specialized training on the assessment device, whereas the control group had no exposure to this equipment. Consequently, the observed intergroup differences may partially reflect task-specific learning effects and advantages from familiarity with the task, consistent with the principle of training specificity [49]. These findings do not entirely represent a fundamental improvement in balance ability. From a potential mechanism perspective, the unstable surface provided by the three-dimensional motion platform, combined with real-time visual guidance adjustments during training, enhances patients’ concentration and balance reaction capabilities. This effect likely stems from the platform stimulating integration within the central sensory nervous system, thereby demonstrating unique advantages in improving balance control, reaction speed, and multitasking abilities [20, 50]. Second, the platform’s advantages also lie in its ability to effectively activate core muscle groups and enhance muscle recruitment for postural control [45], thereby establishing a physiological foundation for limb coordination and improving balance. This finding aligns with conclusions from previous studies [20, 46].Moreover, real-time visual feedback can correct movement deviations and enhance the brain’s control over limbs [51], thereby improving motor performance. This advancement validates the significant advantages of visual feedback balance training, which elevates balance training from simple posture maintenance to a goal-oriented, cognitively engaged motor learning process through the integration of visual feedback. It is noteworthy that the superior rehabilitation outcomes observed in the intervention group were not attributable to a single factor, but rather resulted from the combined effects of multiple mediating factors, including task-specific training, enhanced attentional engagement, and personalized difficulty progression. During training, patients must not only maintain stability but also execute precise center-of-gravity shifts and postural adjustments within that stability, a process that closely simulates the agility and postural control demanded during movement. This training model directly enhances patients’ bodily control in dynamic environments, facilitating efficient recovery of balance function following ACLR.

Pain relief and functional restoration represent fundamental objectives in postoperative rehabilitation following ACLR. Our findings indicate significant improvements in VAS and IKDC scores in the intervention group post-intervention. Additionally, the intervention group demonstrated markedly superior VAS, IKDC, and Lysholm scores compared to the control group. First, visual feedback balance training optimizes the knee’s biomechanical environment by synergistically improving lower limb muscle strength and dynamic balance. The resulting improvement in neuromuscular control and dynamic joint stability likely reduces aberrant loading patterns, thereby contributing to some degree of pain alleviation and facilitating functional recovery [52–54]. Second, traditional balance training may lack objective feedback and personalized progression protocols. It may also fail to effectively address core issues such as muscle strength imbalances, insufficient balance capacity, and pain due to psychological factors [55, 56]. It should be noted that the pain relief observed in this study did not reach the MCID, which may reflect an early trend toward improvement. Since patients who have undergone ACLR are still in the early postoperative phase, their neuromuscular control and motor function have not yet fully recovered; therefore, pain improvement at this stage is limited, suggesting that continued systematic rehabilitation training is necessary to promote further recovery. In summary, this study demonstrates that visual feedback balance training offers certain advantages in enhancing functional performance and shows a positive trend in pain relief [39], which are crucial for ACLR patients to achieve high-quality functional rehabilitation and successful return to sports. However, as sensor-based technologies become increasingly prevalent in rehabilitation, it is crucial to standardize assessment protocols to ensure data validity and reliability across different devices and settings, as emphasized by recent calls for “algorithmic” approaches in sports science [57].

The findings of this study demonstrate that visual feedback balance training outperforms conventional methods in improving muscle strength, balance, and functional outcomes in patients after ACL reconstruction. First, visual feedback provides critical sensory compensation. ACLR surgery often leads to impaired knee joint proprioception, resulting in reduced somatosensory input and neuromuscular control dysfunction. Visual information effectively compensates for this sensory loss, promoting motor control and functional recovery [51, 58]. Second, this training modality constitutes a goal-oriented active motor task. Patients are required to actively control their center of gravity to complete on-screen tasks while receiving real-time visual feedback on postural performance. This “active control-immediate feedback” closed-loop mechanism plays a critical role in enhancing proprioception, improving postural control, and optimizing neuromuscular coordination [44, 58].Notably, the observed improvements in neuromuscular control suggest that adaptive changes may extend beyond the local joint level. Recent studies in patients with chronic ankle instability indicate that localized joint injury can lead to neuromuscular control abnormalities, subsequently affecting proximal joint function-including reduced strength in both knee extensors and flexors [59]. This finding underscores the complexity and multi-joint relevance of post-injury neuromuscular adaptations, suggesting that sensory deficits in a single joint can exert widespread effects on movement control. In the clinical context of anterior cruciate ligament reconstruction, the proprioceptive deficits resulting from ligament injury may similarly extend beyond the knee joint. Interventions such as visual feedback balance training, which enhance multisensory integration, may therefore facilitate more comprehensive neuromuscular functional recovery in patients. In summary, this study provides preliminary evidence supporting the feasibility and effectiveness of integrating visual feedback balance training into early postoperative rehabilitation after ACLR. By offering a structured, task-specific, and real-time feedback environment, this approach enables personalized and precise rehabilitation, offering an innovative strategy to facilitate patients’ safe return to sports.

## Clinical implications

This study provides preliminary evidence supporting the integration of visual feedback-based balance training into early rehabilitation following anterior cruciate ligament reconstruction (ACLR). The findings indicate that this method is a superior alternative to conventional balance training for improving lower limb muscle strength, dynamic postural control, and patient-reported functional outcomes. By providing real-time, objective feedback, it enhances neuromuscular control, promotes limb symmetry, and facilitates targeted motor relearning. Implementing such training from approximately 5 weeks post-surgery can optimize recovery quality, potentially reduce reinjury risk, and support a safer return to sports. Furthermore, the adaptable nature of the technology allows for personalized progression, addressing individual deficits effectively. These results advocate for the adoption of interactive, feedback-driven rehabilitation technologies to advance standard post-ACLR care.

## Limitations

This study has several limitations. First, the final sample size was relatively small, limiting statistical power; therefore, the findings should be considered preliminary and exploratory. Second, dynamic balance was assessed exclusively using the same equipment as the training regimen, potentially introducing device-specific learning effects. Future studies should incorporate independent, clinically validated balance assessment tools (e.g., the Y-balance test). Third, the follow-up period was relatively short, precluding evaluation of long-term rehabilitation outcomes and the risk of re-injury. Fourth, although this study has assessed isokinetic muscle strength at angular velocities of 60°/s and 180°/s, it has not covered higher angular velocities (e.g. 300°/s) and eccentric contraction modes, which to some extent limits the comprehensive assessment of neuromuscular function, high-speed motor performance, and dynamic joint stability. Future studies should expand the sample size, prolong the follow-up time, adopt independent assessment tools, and incorporate a more comprehensive isokinetic test protocol (including high angular velocity and centrifugal mode) to further verify the long-term efficacy and clinical practical value of this rehabilitation protocol.

## Conclusion

This preliminary study suggests that visual feedback balance training may improve lower limb muscle strength, dynamic balance, and functional outcomes in patients after ACLR, though these findings require confirmation in larger, long-term trials. However, the improvements in dynamic balance may partly reflect task-specific proficiency on the assessment device rather than fully generalizing to real-world stability.

## Data Availability

Due to ethical restrictions, the datasets generated during the current study are available from the corresponding author on reasonable request.

## Abbreviations

ACL: Anterior cruciate ligament
ACLR: Anterior cruciate ligament reconstruction
LSI: Limb symmetry index
Qc: Concentric quadriceps strength
Hc: Concentric hamstring strength
H/Q: Hamstring-to-quadriceps strength ratio
VAS: Visual Analogue Scale
IKDC: International knee documentation committee subjective knee form
